# Cutaneous Leukocytoclastic Vasculitis Induced by Apixaban and/or Rivaroxaban With Seronegative Anti-Neutrophil Cytoplasmic Antibody (ANCA) Titers: A Case Report and Literature Review

**DOI:** 10.7759/cureus.44376

**Published:** 2023-08-30

**Authors:** Mohamad El-Sabbagh, Sarah Rifai, Zainalabedeen M Sabah, Adam M Tarakji, Ahmad O Rifai, Sally Dahan, Kristin M Denig

**Affiliations:** 1 Internal Medicine, Alabama College of Osteopathic Medicine, Dothan, USA; 2 Nephrology, The Virtual Nephrologist, Panama City Beach, USA

**Keywords:** lcv, apixaban, rivaroxaban, anca, leukocytoclastic vasculitis

## Abstract

A 45-year-old man presented with a diffuse petechial rash and a non-blanching palpable purpura, mainly on his lower extremities, some of which had coalesced, blistered, and ulcerated. The patient had a history of hypercoagulability and was chronically on anticoagulant medication. The rash appeared a week after starting apixaban 5 mg twice daily by mouth. Prior to that, he was receiving rivaroxaban. The rash was biopsied, which demonstrated cutaneous leukocytoclastic vasculitis (LCV). Serum anti-neutrophil cytoplasmic antibody (ANCA) titers were negative. Complement levels of C3, C4, and CH50 were normal. Hepatitis C antibodies were negative. HIV antibodies were non-reactive. Titers for Lyme disease and Rocky Mountain spotted fever were nonreactive. It is unusual for a drug to induce cutaneous LCV with negative ANCA titers. Although rare, it usually requires aggressive therapy. Our case resolved after the discontinuation of apixaban and rivaroxaban and the initiation of warfarin for hypercoagulability in conjunction with a short course of steroids. As the use of apixaban and rivaroxaban increases, we may see a consequent increase in cutaneous LCV that is specifically ANCA-negative.

## Introduction

Cutaneous leukocytoclastic vasculitis (LCV) is a histopathologic term that describes angiitis of the cutaneous small blood vessels limited to the skin, where the infiltrate is predominantly composed of neutrophils [1]. Following degranulation of neutrophils, they undergo cell death and rupture through a process termed leukocytoclasia, consequently releasing nuclear dust [1]. It is reported that the annual incidence of biopsy-proven cutaneous LCV is 38.6 per million [1]. The etiology of cutaneous LCV is primarily idiopathic, leading to the designation' primary cutaneous LCV [2]. However, it can also be secondary to various causes: infections such as hepatitis B, hepatitis C, syphilis, or HIV; autoimmune disorders like Sjogren's Syndrome, Crohn's disease, rheumatoid arthritis, or mixed connective tissue disorder (MCTD); and malignancies such as lymphomas or lymphoproliferative disorders. Among these, drug reactions are the most common causes of secondary cutaneous LCV [2-3]. While cutaneous LCV can be part of a systemic vasculitis syndrome, it can also be associated with cryoglobulins or paraproteinemia [1]. Signs of cutaneous LCV typically appear about 1-3 weeks after the triggering event [4]. These include erythematous macules with palpable purpura, predominantly found bilaterally on the lower extremities. These lesions can sometimes coalesce and even ulcerate. While rarer presentations might be localized or unilateral [4], the rash can also, to a lesser extent, appear on the upper extremities or the trunk [5]. The diagnosis of LCV is established through a skin biopsy, complemented by direct immunofluorescence [6]. We present a case of a patient with a history of hypercoagulability. He had been treated with rivaroxaban for several months. However, within a week of switching to apixaban, he developed a diffuse petechial rash and palpable purpura on his lower extremities, with a few faint scattered lesions on his upper extremities. A biopsy of the rash on his right lower extremity confirmed LCV. Notably, his anti-neutrophil cytoplasmic antibody titers and other markers associated with cutaneous LCV were negative.

## Case presentation

**Figure 1:**
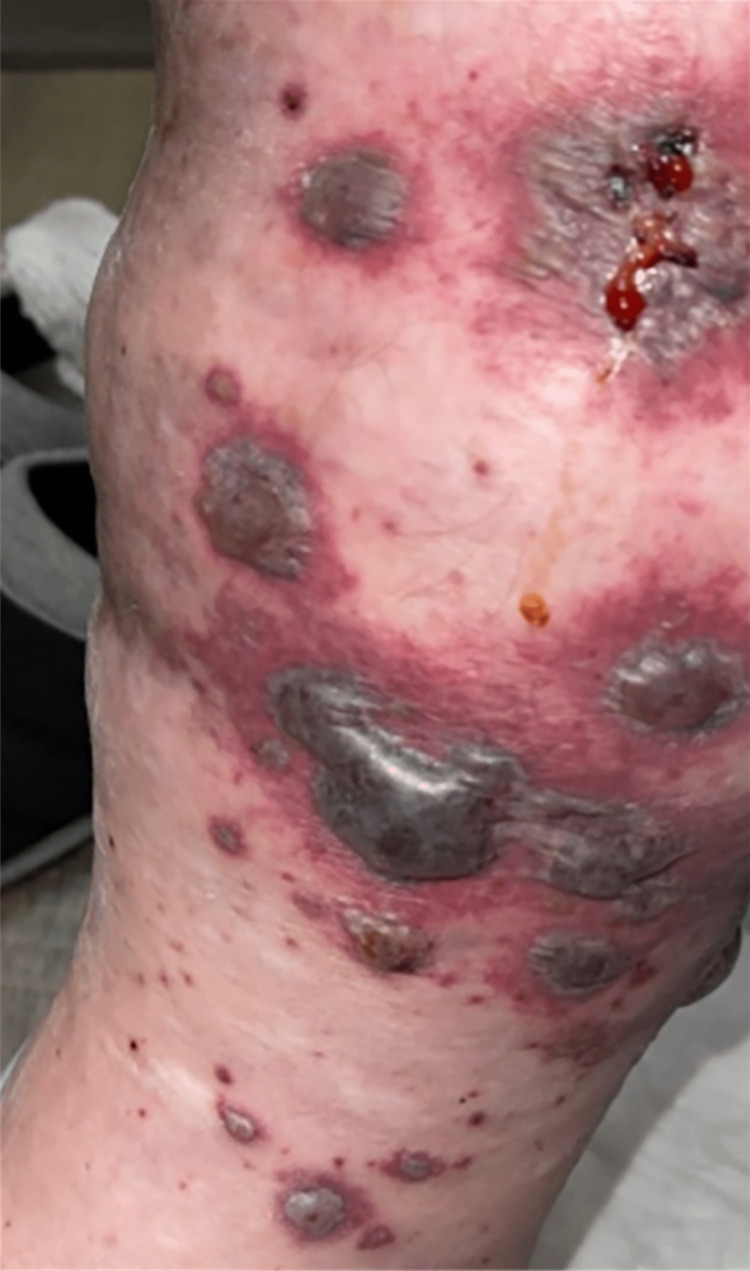
Lower extremity.

**Figure 2:**
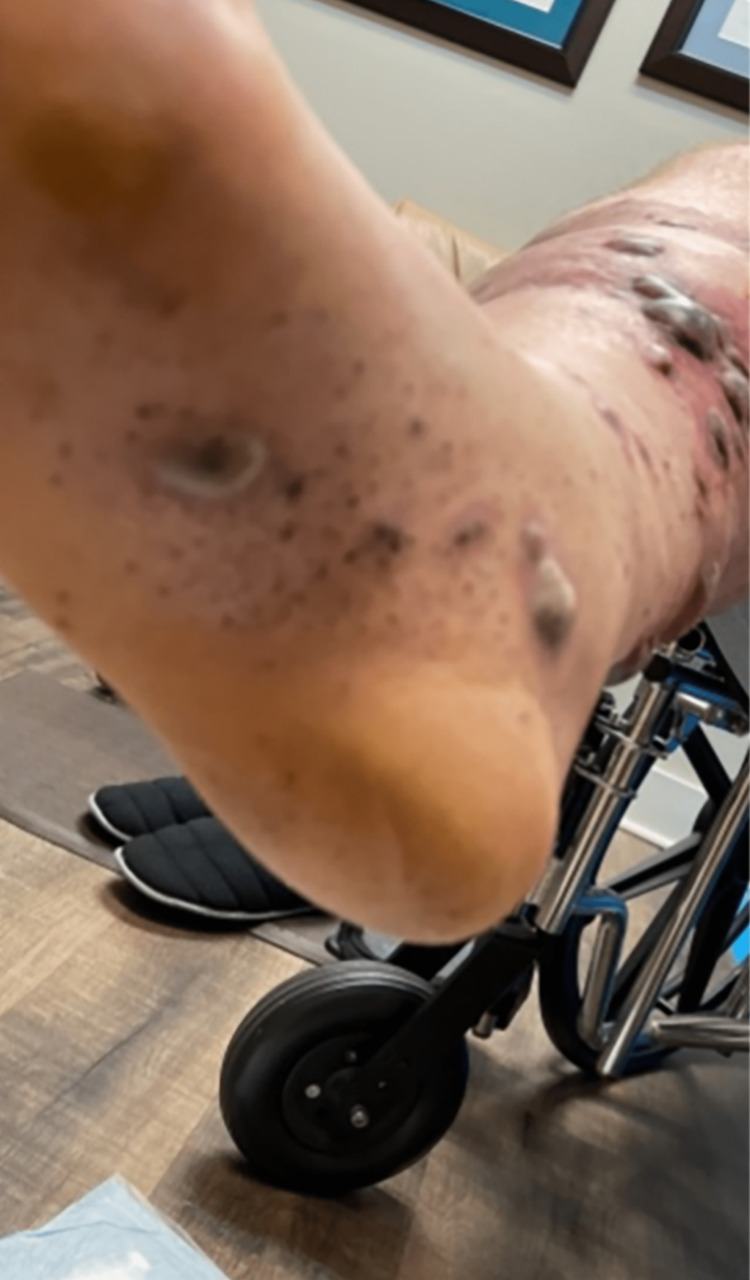
Lower extremity.

**Figure 3:**
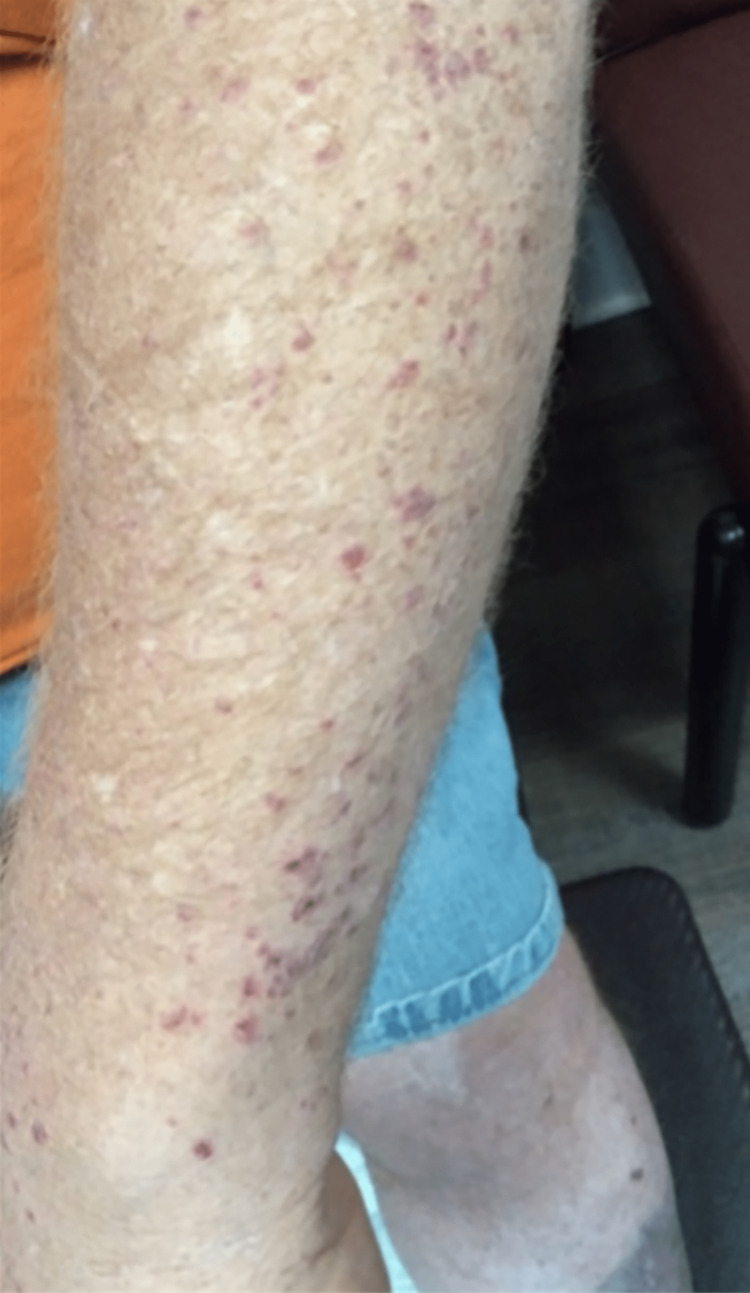
Upper extremity.

A 45-year-old morbidly obese male was referred from his vascular surgeon’s office due to a new diffuse petechial rash with bullae over the lower extremities, raising concerns for vasculitis. He had a past medical history of hypertension, gout, and recurrent deep vein thrombosis (DVTs) spanning the previous 20 years. Upon evaluation in the ED, his ECG showed atrioventricular dissociation accompanied by bradycardia. Further, he exhibited renal insufficiency with an elevated serum creatinine level of 1.8 mg/dL, proteinuria, and hematuria. Bilateral lower extremity edema was also observed. The patient reported that his rash began a month prior to his presentation and started a week after switching from rivaroxaban to apixaban. He had been on apixaban for only one week when the rash appeared. Despite the discontinuation of apixaban, the rash persisted. He also mentioned a history of recurrent DVTs over the past 20 years and had experienced a pulmonary embolism leading to an inferior vena cava (IVC) filter placement. Due to discomfort in his lower extremities, he regularly took ibuprofen and was on gabapentin, 300 mg 2-3 times daily. He was also prescribed lisinopril. Physical examination revealed a blood pressure of 121/97 mmHg, heart rate of 37 beats per minute (bpm), temperature of 36.8°C, and a respiratory rate of 18 breaths per minute. The patient was alert, oriented, and appeared non-toxic. He was pleasant and conversant. Cardiovascular examination revealed distant heart sounds, attributed to the patient's body habitus, and bradycardia with a regular rhythm. The skin examination showed a petechial rash on both the upper and lower extremities (Figures 1-3), including the palms and soles. Large confluent areas of purpura were observed bilaterally on the lower extremities, with occasional sizable bullae.

The laboratory investigations yielded specific results, as detailed in Table 1.

**Table 1:** Laboratory findings.

Lab results	Range
WBC count	
16.8 K/µL	4.5-11.0 K/µL
Hemoglobin	
13.5 g/dL	12.0-15.5 g/dL
Hematocrit	
41.1%	34.9-44.5%
Serum Creatinine	
1.8 mg/dL	0.7-1.3 mg/dL
Blood urea nitrogen (BUN)	
17.0 mg/dL	7-20 mg/dL
Glucose	
122 mg/dL	70-99 mg/dL
Anti-nuclear antibodies (ANA) screen	
Negative	N/A
ANCA titers	
<1:20	N/A
C3 complement	
139.7 mg/dL	82-185 mg/dL
C4 complement	
16.3 mg/dL	15-53 mg/dL
Complement activity, total turbidimetric	
74.8 unit/mL	38.7-89.9 unit/mL
Erythrocyte sedimentation rate (ESR)	
15 mm/hour	0-22 mm/hour
Cryoglobulin, Qualitative	
Negative at 72 hours	<30 IU/mL
Anti-dsDNA antibodies	
0.9 IU/mL	<9.9 IU/mL
Hepatitis C antibodies	
Non-reactive	N/A
Human Immunodeficiency Antigen and Antibodies	
Non-reactive	N/A

**Figure 4:**
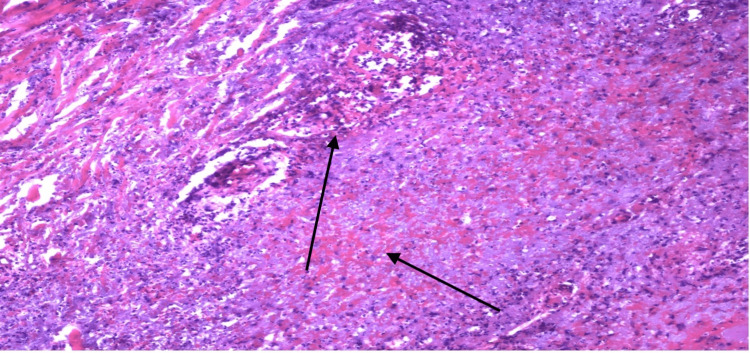
20X view. The center of the image displays a degenerated, relatively large blood vessel interspersed with numerous neutrophils and evident fibrinoid necrosis of the vascular wall. To the right of the vessel, there are abundant extravasated red blood cells. The small dark dots, abundant throughout the image, represent fragmented neutrophil nuclei, indicative of leukocytoclasia.

**Figure 5:**
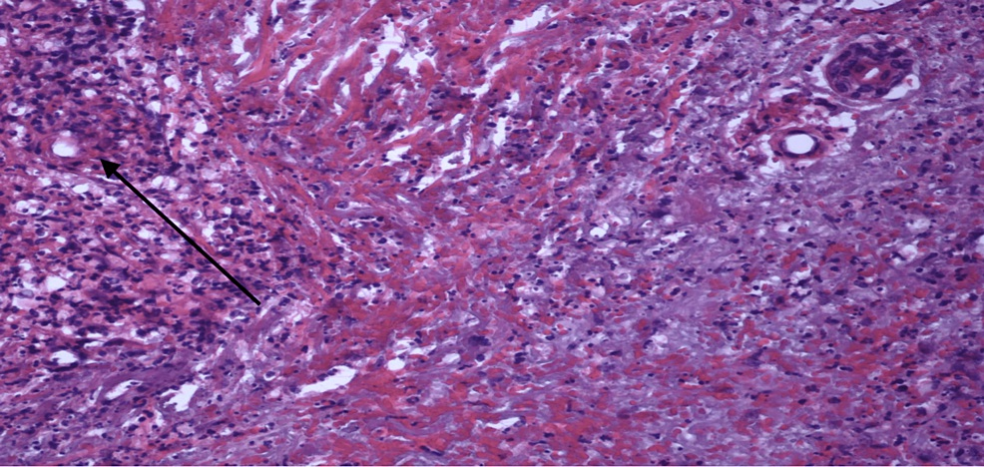
20X view. The top left reveals numerous neutrophils targeting at least one small vessel. Throughout the panel, there's a noticeable presence of extravasated red blood cells and abundant leukocytoclasia.

**Figure 6:**
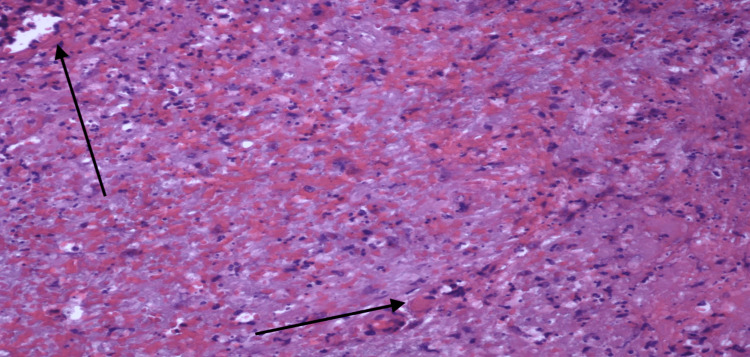
20X view. The image showcases abundant leukocytoclasia and extravasated red blood cells throughout. Scattered small vessels exhibiting leukocytoclastic vasculitis are also evident.

The findings of the skin biopsy are described in Figures 4-6.

## Discussion

Systemic LCV, characterized by neutrophil infiltration of blood vessels, is typically associated with positive ANCA titers [7-8]. However, in cutaneous LCV, this association is not as pronounced; in this case, the patient's ANCA titers were negative. Additionally, some instances of cutaneous LCV are linked with systemic lupus erythematosus (SLE) [7]. The patient's complement levels (C3, C4) were within the normal range, and his anti-dsDNA tests came back negative, ruling out active SLE. Although hepatitis C can present with vasculitis [1], the patient tested negative for hepatitis C. The only diagnostic test confirming cutaneous LCV was the skin biopsy of the lesions. Our diagnosis pointed to either primary cutaneous LCV or drug-induced cutaneous LCV. Given the absence of positive laboratory test results, such a presentation is rare. The patient’s lesions resolved shortly after the discontinuation of apixaban and rivaroxaban, aided by a brief regimen of oral steroids. Another noteworthy observation is that our patient had normal eosinophil levels, registering at 0.7%. A review of the literature did not identify a clear relationship between blood eosinophilia and drug-induced cutaneous LCV. According to Bahrami S et al. [9], tissue eosinophilia in patients with cutaneous LCV is typically elevated. It has been established that tissue eosinophilia is a reliable indicator of drug-induced cutaneous LCV [9].

In our case, no kidney biopsy was performed to confirm a possible diagnosis of glomerulonephritis or capillaritis despite the patient presenting with elevated serum creatinine, proteinuria, and hematuria. Consequently, whether this cutaneous LCV was part of a systemic LCV remains uncertain. The absence of positive P-ANCA or C-ANCA titers did not support the decision to conduct a kidney biopsy. Additionally, the procedure would have been technically challenging due to the patient's morbid obesity and ongoing full anticoagulation therapy. In addition, the patient’s elevated creatinine was not new and had been stable for months, according to his records. Immunofluorescent testing was not performed because we are a small community hospital, and the diagnosis was evident based on the H&E staining. Another limitation we faced was the timing of rivaroxaban and apixaban initiation and discontinuation. It was difficult to determine the exact cause in our case. The cutaneous LCV could have resulted from rivaroxaban and manifested around the time apixaban was initiated, or it might have been directly caused by the introduction of apixaban. Another possibility is that this might have been an idiopathic or primary case that coincidently happened to present around the time of initiation of this new anticoagulative medication since all ANCA titers were negative. A literature review reveals two cases of cutaneous LCV with apixaban and two cases of cutaneous LCV with rivaroxaban. In the apixaban cases, one was ANCA negative [10], and in the second case, ANCA status was never reported [11]. The ANCA status was not reported for the two cases of rivaroxaban [12,13]. However, the Hasbal NB et al. report [12] mentioned that all rheumatological workup was negative and the patient had IgA nephropathy, although ANCA titers were not specified as a part of the workup. It is not clear whether these cases were ANCA-positive or not. For other cases of drug-induced cutaneous LCV, they usually have had P-ANCA to be a lot more common than C-ANCA. Weng CH and Liu ZC [8] noted no clear epidemiological data to determine the incidence of drug-induced ANCA-associated vasculitis. Our case may represent the third known instance related to either apixaban or rivaroxaban. There is limited information about the association of ANCA with cutaneous LCV due to apixaban or rivaroxaban. In contrast, drugs like Methimazole and Hydralazine have been studied for a more extended period and are frequently ANCA-positive in instances of drug-induced cutaneous LCV. It remains unclear whether the absence of ANCA in drug-induced cutaneous LCV results from a distinct allergic pathway or is specifically attributed to the medications apixaban or rivaroxaban.

## Conclusions

Our case has demonstrated that cutaneous LCV can be triggered by apixaban and/or rivaroxaban. They are relatively new medications and are being used with increasing frequency. Although it is unusual to have negative ANCA with classic biopsy-proven drug-induced cutaneous LCV, our patient had a biopsy-proven cutaneous LCV and negative titers for ANCA. The absence of ANCA may be the classic presentation of apixaban or rivaroxaban-induced cutaneous LCV. As the use of apixaban or rivaroxaban increases, more related cases of cutaneous LCV may answer this dilemma.
